# Commit* to change? A call to end the publication of the phrase ‘commit* suicide’

**DOI:** 10.12688/wellcomeopenres.10333.1

**Published:** 2016-12-06

**Authors:** Emma Nielsen, Prianka Padmanathan, Duleeka Knipe

**Affiliations:** 1 Self-Harm Research Group, School of Psychology, University of Nottingham, Nottingham, UK; 2 School of Social and Community Medicine, University of Bristol, Bristol, UK

**Keywords:** suicide, self-harm, stigma

## Abstract

**Background.** Countering stigma is a fundamental facet of suicide prevention efforts.  Integral to this is the promotion of accurate and sensitive language.  The phrase ‘commit* suicide’ has prompted marked opposition primarily due to the connotations of immorality and illegality. **Methods.** The study investigated the frequency of the use of the wordstem ‘commit’, in relation to self-harm and suicidal behaviours, in the three leading suicide-specific academic journals between 2000 and 2015. **Results. **One third (34%) of articles published since the year 2000 used the word ‘commit*’ when describing an act of self-harm or suicide. Over half of these articles (57%) used the phrase on more than one occasion, with 6% using it more than 10 times in the same manuscript. The percentage of papers utilising the word ‘commit*’ has fluctuated over time, but there is a promising downward trend in the use of this phrase from 33% in 2000 to 13% in 2015 ( *p* < 0.001). **Discussion.** We advocate for the implementation of publication requirements regarding the language used when discussing suicide. Whilst we call for collective responsibility amongst academics and clinicians, editors hold a unique position in ensuring that outdated, inaccurate and stigma-laden terms are expunged from the scientific literature.

## Introduction

Suicide is a complex, multifaceted behaviour indicative of psychological distress ( Williams & Pollock, 2001), which is also highly stigmatised ( Cvinar, 2005). This is of concern, given that mental health stigma impacts negatively upon both help-seeking behaviours and help-provision ( Corrigan *et al.*, 2003; Reynders *et al.*, 2014). Consequently, a fundamental facet of suicide prevention efforts must be the eradication of stigma. Integral to this is the promotion of sensitive language that is accurate, non-divisive and does not propagate unhelpful connotations.

### Implications of language - immorality and illegality?

*“We now live in a time when we seek to understand people who experience suicidal ideation, behaviours and attempts, and to treat them with compassion rather than condemn them.”* ( Beaton *et al.*, 2013).

While a range of potentially unhelpful terminology has been identified (e.g., Silverman, 2006), the phrase ‘commit* suicide’ has perhaps prompted the most publicised opposition (to aid readability, ‘commit* suicide’ will be noted as ‘commit suicide’ from here on in. The comments made, however, extend to all suffixes of the stemword when used to discuss suicide). Although ‘commit’ has a number of meanings, the phrase is most commonly associated with negativity and wrong-doing ( Beaton *et al.*, 2013; Silverman, 2006); people *commit* crimes, people *commit* moral atrocities. 

A recent global review of the legal status of suicide indicated that in the majority of countries (and states) suicide is not a crime; of the 192 criminal codes obtained, suicide was illegal in only 25 countries (with a further 20 countries adhering to Sharia or Islamic law) ( Mishara & Weisstub, 2016). Most nations have updated their legislation to decriminalise suicidal behaviour. However, commonly employed nomenclature does not always reflect this. As well as carrying connotations of illegality, the phrase ‘commit suicide’ can also suggest immorality or dishonour ( Silverman, 2006). While opinions regarding suicide vary, it is unusual to associate the term ‘commit’ with a public health concern, or indeed a mental health tragedy. Further, this phraseology does not acknowledge the psychological distress associated with suicidality ( Silverman, 2006).

### The impact of language

*“So, to say that someone “committed” suicide feels offensive to me, and I’m not easily offended. The offense is in the inaccuracy.”* (Kyle Freeman, 2015).

Arguably our language should reflect the needs and experiences of those directly affected by suicide. Indeed for many it can be insensitive language that amplifies the distress of an already difficult situation, increasing feelings of blame, guilt and rejection (Kyle Freeman, 2015, https://themighty.com/2015/07/why-you-shouldnt-say-committed-suicide/; Maple *et al.*, 2010; Silverman, 2006; Sommer-Rotenberg, 1998); the negative connotations inherent in stigmatised language may inadvertently exacerbate vulnerabilities ( Cvinar, 2005).

### Committed to change? Publication guidelines

In academic literatures there is a precedent for demanding considered language and the eradication of stigmatised phraseology from published parlance. For example, the American Psychological Association editorial guidelines caution against referring to people in diagnostic terms, advocating instead for the adoption of ‘people first’ language (e.g., ‘ *people who* self-harm’, rather than ‘self-harmers’; ‘200 *people who* took their lives by hanging’, rather than ‘200 suicides by hanging’) ( American Psychological Association, 2010). Subject specific guidelines have also been published by some journals, highlighting problematic terms and favoured alternatives (e.g., terms to avoid or reconsider in the eating disorders field; Weissman *et al.*, 2016).

Considering suicide, outside of academia there are a number of guidelines for the reporting and discussion of suicide (e.g., Hunter Institute of Mental Health, 2014; Samaritans, 2013; World Health Organization, 2008). Many of these media frameworks explicitly highlight the difficulties of the phrase ‘commit suicide’ (e.g., World Health Organization, 2008). While some researchers have highlighted that the term ‘commit suicide’ should be considered within “possible terms to be removed from the lexicon” ( Silverman, 2006), to the best of our knowledge, there are currently no explicit guidelines, or indeed requirements, in place regarding phraseology in academic publication.

### Current study

The phrase ‘commit suicide’ is a pervasive term. As researchers, clinicians and suicidologists, we arguably hold a collective responsibility to lead by example, to take heed of the extant literature and to listen to the voices of those with lived experience and expertise. The current study seeks to explore whether the marked opposition to the term ‘commit suicide’ is reflected in a decrease or indeed abolition of its publication. Specifically, the study aims to investigate whether:

(i) The phrase ‘commit suicide’ is still being published in the three leading suicide-specific academic journals;
(ii) There have been reductions in the use of the stemword ‘commit’ in relation to acts of self-harm and/or suicide.

## Methods

We investigated the frequency of use of the wordstem ‘commit’, in relation to self-harm and suicidal behaviours, in three high impact suicide-specific academic journals between 2000 and 2015. We assessed the trend during this time period, as well as differences in the frequency of use between the journals.

### Inclusion and exclusion criteria

We selected the three leading academic journals on suicide and suicide prevention: Crisis, Archives of Suicide Research (ASR) and Suicide and Life-Threatening Behavior (SLTB). These journals were chosen because they: i) have a long history of publishing articles in the field of suicidology (at least 20 years); and ii) represent the peer-reviewed journals associated with the two main international associations of suicide researchers. We included all article types within all issues of each journal between 2000 and 2015. Journal issues prior to 2000 were inaccessible online and therefore excluded. Archives of Suicide Research did not publish issues in 2000 and 2001.

### Data collection

Each author collected data from a single journal using a standardised data extraction form. For each article within an issue, we collected data on the number of times the word stem ‘commit’ was used outside quotation marks or inverted commas in the title, abstract and main text. Data for all article types were extracted, including editorials, letters, book reviews (etc.), as well as empirical papers, reviews and meta-analyses.

### Statistical analysis

To adjust for the number of articles per journal each year, we tabulated and graphed the frequency of the word stem ‘commit’ as a percentage of the total papers in each year. All analysis was conducted in Stata 14 (StataCorp, College Station, TX). We also tested to see whether there was evidence of a linear reduction, with a Chi-squared test for linear trend, in the use of the phrase ‘commit suicide’ overall.

## Results

There were 2298 articles published in the three main suicide related journals since the start of the millennium (2000–2015). Of these, 781 (34%) articles used the word ‘commit’ when describing an act of self-harm or suicide. Over half of these articles (57%) used the word ‘commit’ in more than one instance in the same paper, with 6% using it more than 10 times in the same manuscript.

**Figure 1.**
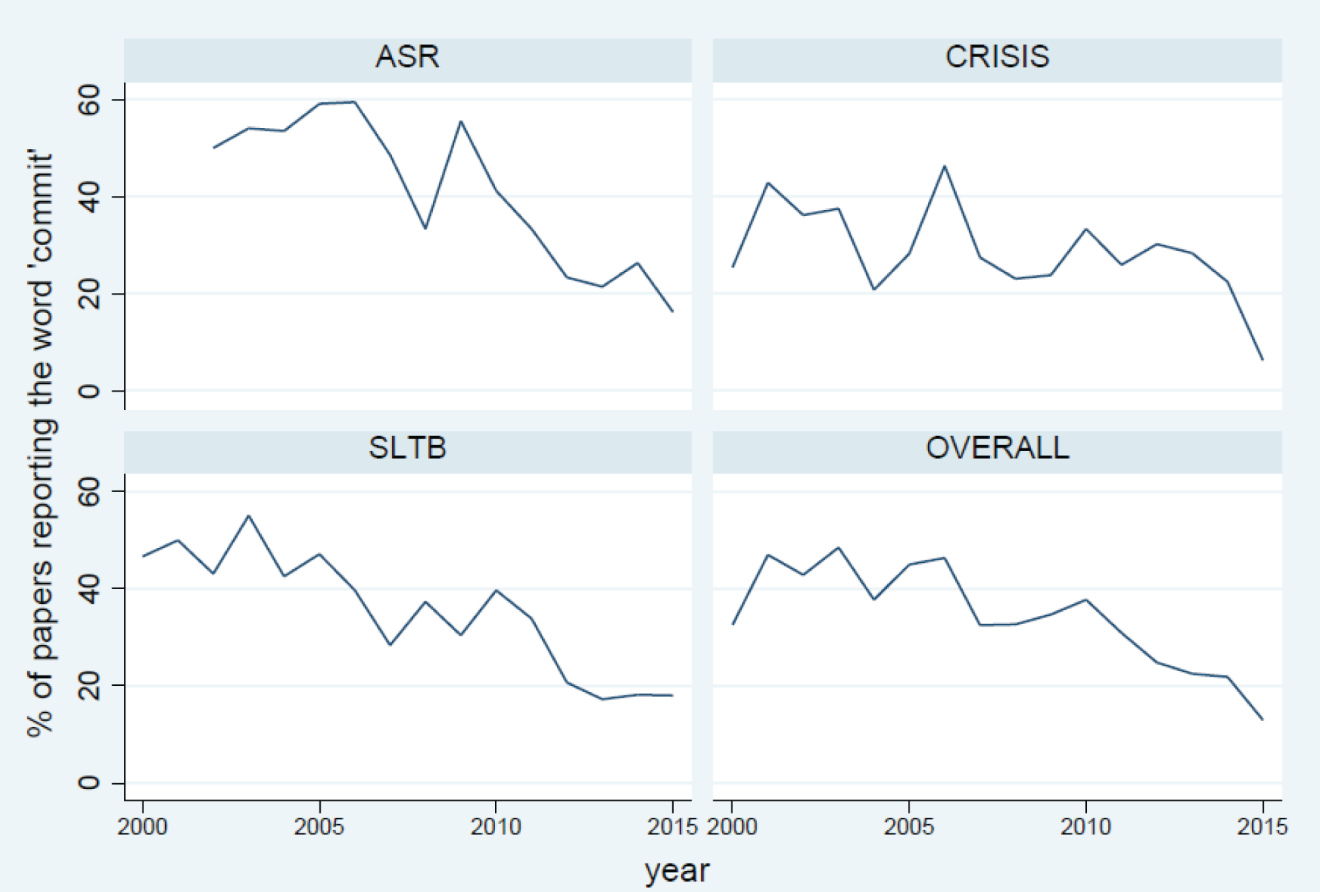
Percentage of papers reporting ‘commit*’ in relation to suicide or self-harm behaviours, by journal (2000–2015), *p*-value for trend < 0.001. Note: Instances in which ‘commit*’ is directly quoted (e.g., in qualitative interview data that is reported to support a theme) are excluded from this count. ASR, Archives of Suicide Research; SLTB, Suicide and Life-Threatening Behaviour.

The percentage of papers utilising the word ‘commit’ has fluctuated over time, but in all journals included in this review there has been a reduction in the use of this word ( Figure 1; *p*-value for trend < 0.001). In the most recent year (2015) across all three journals, 13% of articles use the word ‘commit’ (SLTB, 18%; CRISIS, 6%; and ASR, 16%).

## Discussion

In all three of the leading suicide-specific academic journals, the phrase ‘commit suicide’ continues to be published. Reassuringly there have been reductions in the use of this term in relation to acts of self-harm or suicide. However, in 2015 one in eight articles still used this outdated, largely inaccurate and stigmatised phrase.

The International Association for Suicide Prevention (IASP) collaborated with the World Health Organization (WHO) to produce the 2008 revision of the ‘preventing suicide’ framework for media professionals ( World Health Organization, 2008). This resource specifies that “the phrase ‘committed suicide’ should not be used because it implies criminality, thereby contributing to the stigma experienced by those who have lost a loved one to suicide and discouraging suicidal individuals from seeking help” (p. 8). It is therefore perhaps particularly surprising to note the continued publication of the phrase ‘commit suicide’ since 2008 in Crisis, the journal of IASP.

As individuals we each hold a position of power and responsibility. As researchers, clinicians and suicidologists we hold a collective responsibility for the language we propagate and the messages this imparts. Therefore, we believe that the phrase ‘commit suicide’ should be avoided in all arenas, including clinical and professional practice, teaching, when interacting with peers, colleagues and the general public, in our everyday lives and, importantly, in our academic writing. While it is noted that in the 25 countries in which suicide remains illegal, the use of phrase is perhaps more understandable, it is of paramount importance that we hold ourselves to the standards to which we seek to hold others (e.g., journalists, politicians, opinions leaders). Much as suicide-specific media guidelines advocate for the consideration of appropriate language choices, we believe that: i) journals should include in their author guidelines a note necessitating appropriate language; and ii) journal reviewers should take responsibility in insisting that language is amended if inappropriate.

Suicide is a cause of death and our language should reflect this; people *die by* suicide. Guidelines necessitating the abolition of the phrase ‘commit suicide’ (in non-quoted prose) may be one step towards facilitating this goal. Indeed, editors hold a unique position in ensuring that outdated, inaccurate and stigma-laden terms with criminal overtones are expunged from the scientific literature. Addressing the language we use affords an important opportunity to meaningfully contribute to the eradication of the stigma surrounding suicidality.

This study considered only instances in which the phrase ‘commit suicide’ was used outside of quotation marks or inverted commas. This inclusion criteria was imposed for two primary reasons: i) we believe that individuals have the right to describe their own experiences in their own terms; therefore, particularly in qualitative or mixed methods papers, the stemword ‘commit’ may appear appropriately within a verbatim quotation from a participant; and ii) the phrase ‘commit suicide’ may be included within quotation marks in papers highlighting the problematic nature of this language (e.g., Silverman, 2006). Indeed the phrase ‘commit suicide’ frequently appears within inverted commas throughout this article. Future research may seek to explore trends in the frequency and nature of use of the stemword ‘commit’, in relation to self-harm and suicide, within quoted prose.

We hope that the current study will act as a catalyst for discussion and prompt further consideration of: i) the inherent connotations of the language frequently employed within in the field; ii) how we may inadvertently perpetrate stigma; and iii) how we might work to eradicate this stigma. This will only be achieved by working collaboratively with those with lived experience.

### Limitations

The study must be interpreted within the context of its limitation. Herein, it is important to note that ‘commit’ is not the only potentially difficult language propagated in the literature. For example, in the United Kingdom the Royal College of Psychiatrists have recently updated their language around self-harm (‘deliberate self-harm’ to ‘self-harm’) in light of concerns highlighted by mental health service consumers and those with lived experience of self-harm ( Silverman, 2006). A number of other potentially difficult phrases persist within academic communications (e.g., labelling language such as ‘self-harmers’, ‘ideators’, ‘jumpers’, phrases such as ‘copy-cat’) against a backdrop of non-standardised and non-agreed nomenclature. Indeed determining appropriate and unhelpful terms will require review and augmentation over time.

The current study examined trends since the start of the millennium in the three leading suicide-specific academic journals. The study examined all online material published within this 15 year period. However, it is important to note that suicide is covered across a range of journals and fields (e.g., psychology, sociology, psychiatry, epidemiology, public health), each of which will have established traditions. While we argue for the role of dedicated suicidology journals, researchers and clinicians leading by example, to make meaningful and lasting change further work must consider how guidelines could translate across disciplines and platforms (e.g., consideration must be given to the language propagated in academic proceedings, press releases and at conferences).

Notwithstanding these limitations, the present study offers novel insight into recent trends in the publication of the stemword ‘commit’ used in relation to self-harm and suicide. Given the marked opposition expressed in regards to this phraseology, it is perhaps surprising to see the continuation of its publication across the three leading suicide-specific academic journals and indeed propagated in academic proceedings and conferences. Our results suggest that the introduction of author guidelines surrounding language use may be necessary to ensure the abolition of outdated, inaccurate and stigma-laden terms and the adoption of preferred phraseology.

## Data availability

Available online via the Open Science Framework: https://osf.io/br8jd/; DOI, 10.17605/OSF.IO/BR8JD ( Knipe, 2016).
