# Supplementary material for: Commit* to change? A call to end the publication of the phrase ‘commit* suicide’
Source: Wellcome Open Res. 2016 Dec 6;1:21. [Version 1] doi: 10.12688/wellcomeopenres.10333.1 (PMC5341764; doi:10.12688/wellcomeopenres.10333.1)
Supplement: Peer review history

# Peer review: Referee response for version 1

**DOI:** 10.21956/wellcomeopenres.11132.r20483

**Published:** 2017-02-23

**Authors:** Sarah Rowe

**Affiliations:** 1 Institute of Psychiatry, Psychology & Neuroscience, King's College London, London, UK

The title and abstract are appropriate and accurately reflect the work that has been conducted. The design and methods are suitable and the results adequately discussed. The implications of the study are reasonable and balanced. I would like to see a sentence or two in the discussion which proposes alternatives to the phrase ‘commit suicide’ as suggested by people with lived experience and clinicians/researchers. Overall, this paper is interesting and highlights an important issue in need of progression.

I have read this submission. I believe that I have an appropriate level of expertise to confirm that it is of an acceptable scientific standard.

# Peer review: Referee response for version 1

**DOI:** 10.21956/wellcomeopenres.11132.r19042

**Published:** 2017-02-01

**Authors:** Katie Dhingra

**Affiliations:** 1 Department of Criminology, Leeds Beckett University, Leeds, UK

Through a careful and considered review of the previous literature, the authors offer a strong argument for limiting the use of negatively associated language (i.e., “committed”), and instead using language that accurately and sensitively describes experience, such as, ‘died by suicide’.

 The study design as outlined in the Methods section is clear and concise, as is the Results section. The results encouragingly indicate that there has been a reduction in the use of the word “commit” in the three main suicide-specific journals but that there is still more work to do to ensure the stigma associated with suicide is not added to.

 The authors indicate that they hope their study will act as a catalyst for discussion and further consideration of the words that we use, and the ways in which we say things. This is a worthy goal as a simple switch of one word to another could eradicate any connotations of criminality, shame or sin, thus destigmatising the deceased as well as those bereaved by suicide. The authors’ suggestion that editors and reviewers could play an important role in encouraging the use of more appropriate language is a good one. I hope that this is something they have, or intend to pursue directly with the Editor-in-Chiefs of the journals that form the basis of this study (at a minimum).

 A few minor points to consider:

1.  “In academic literatures there is a precedent for demanding considered language…” – the word ‘demanding’ seems too strong in this context.
2. Aim 1 – seems a little redundant given Aim 2
3. “In 2015 one in eight articles still used this outdated, largely inaccurate and stigmatised phrase” – is it possible to consider this phrase truly outdated if we are still using measures that use the phrase?

I have no other comments to make that I believe would strengthen this paper.

I have read this submission. I believe that I have an appropriate level of expertise to confirm that it is of an acceptable scientific standard.
